# Supplementary material for: The Role of Temperature on the Degree of End-Closing and Filling of Single-Walled Carbon Nanotubes
Source: Nanomaterials (Basel). 2021 Dec 11;11(12):3365. doi: 10.3390/nano11123365 (PMC8704686; doi:10.3390/nano11123365)
Supplement: Supplementary file 1 [file nanomaterials-11-03365-s001.zip › nanomaterials-1474217-supplementary.pdf]

nanomaterials

Supplementary Material

The Role of Temperature on the Degree of End-Closing and Filling of Single-Walled Carbon Nanotubes

Magdalena Kierkowicz [1], Elzbieta Pach [2], Julio Fraile [1], Concepción Domingo [1], Belén Ballesteros [2] and Gerard Tobias [1,*]

[1] Institut de Ciència de Materials de Barcelona (ICMAB-CSIC), Campus UAB, 08193 Bellaterra, Barcelona, Spain; mkierkowicz@gmail.com (M.K.); julio@icmab.es (J.F.); conchi@icmab.es (C.D.)
[2] Catalan Institute of Nanoscience and Nanotechnology (ICN2), CSIC and the Barcelona Institute of Science and Technology, Campus UAB, 08193 Bellaterra, Barcelona, Spain; epach@icmab.es (E.P.); belen.ballesteros@icn2.cat (B.B.)
* Correspondence: gerard.tobias@icmab.es

Steam treatment

As-received SWCNTs, containing a fraction of DWCNTs, were finely ground in an agate mortar with a pestle, loaded into a silica tube end exposed to steam under Ar atmosphere at 900 °C during 4 h. Then, they were refluxed with 6 M HCl (PanReac AppliChem) at 110 °C overnight. The resulting sample was rinsed with distilled water until neutral pH, filtered onto a polycarbonate membrane (Whatman Cyclopore, pore size 0.2 μm) and dried in an oven at 100 °C overnight.

Nitrogen adsorption-desorption experiments

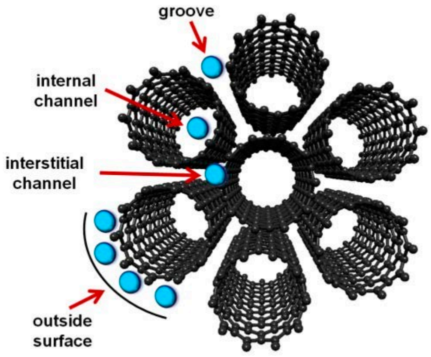

Figure S1. Schematic representation of gas adsorption in a bundle of SWCNTs. The blue dots represent the gas molecules adsorbed on accessible surfaces.

Table S1. Apparent specific surface area (S_{BET}) and cumulative pore volume up to 1.5 nm pore diameter (V_C) of as-received and empty purified/annealed samples. Data obtained from low temperature nitrogen adsorption and NLDFT calculations.

Sample	S_{BET} [m^{2}g^{-1}]	V_C [cm^{3}g^{-1}]
As-received	1246	0.146
RT	1263	0.231
400 °C	1285	0.220
700 °C	1196	0.221
900 °C	1157	0.199
1100 °C	791	0.194

Raman spectroscopy

Table S2. I_D/I_G Raman ratio of purified and annealed samples. Each value is averaged based on three measurements. CNTs were excited using 532 nm laser wavelength.

Sample	I_D/I_G	Error
RT	0.13	0.03
400 °C	0.11	0.03
700 °C	0.13	0.01
900 °C	0.14	0.01
1100 °C	0.12	0.02

Electron microscopy

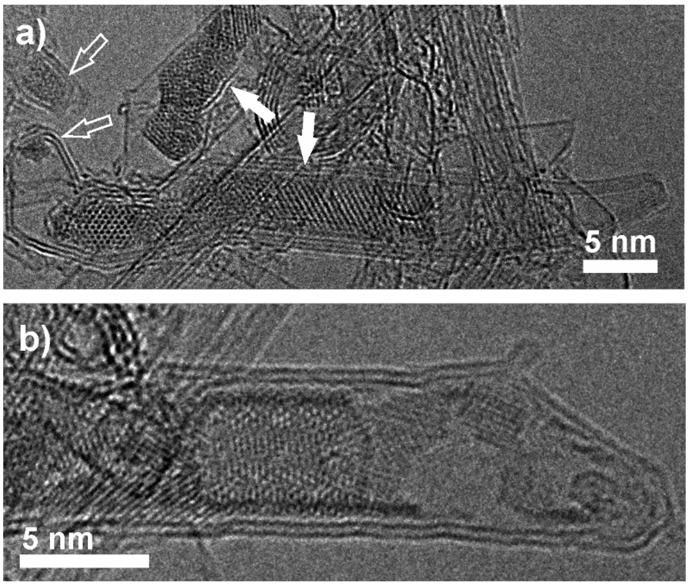

Figure S2. Additional HRTEM images of $EuCl_3$@CNTs prepared at 1100 °C. As a guide to the eye, white arrows point to CNTs filled with nanowires of $EuCl_3$, empty white arrows to filling with nanoparticles, and black arrows point to closed ends. A single-layered inorganic nanotube of $EuCl_3$ was found inside the cavities of a large diameter DWCNT (Figure S2b), thus creating a tubular (1 D) van der Waals heterostructure,
